# Histone H3 and H4 Modifications Point to Transcriptional Suppression as a Component of Winter Freeze Tolerance in the Gall Fly *Eurosta solidaginis*

**DOI:** 10.3390/ijms241210153

**Published:** 2023-06-15

**Authors:** Tighe Bloskie, Kenneth B. Storey

**Affiliations:** Institute of Biochemistry and Department of Biology, Carleton University, 1125 Colonel By Drive, Ottawa, ON K1S 5B6, Canada; tighebloskie@cmail.carleton.ca

**Keywords:** histone modification, epigenetics, freeze tolerance, hypometabolism, goldenrod gall fly

## Abstract

The goldenrod gall fly (*Eurosta solidaginis)* is a well-studied model of insect freeze tolerance. In situations of prolonged winter subzero temperatures, larvae of *E. solidaginis* accept ice penetration throughout extracellular spaces while protecting the intracellular environment by producing extreme amounts of glycerol and sorbitol as cryoprotectants. Hypometabolism (diapause) is implemented, and energy use is reprioritized to essential pathways. Gene transcription is one energy-expensive process likely suppressed over the winter, in part, due to epigenetic controls. The present study profiled the prevalence of 24 histone H3/H4 modifications of *E. solidaginis* larvae after 3-week acclimations to decreasing environmental temperatures (5 °C, −5 °C and −15 °C). Using immunoblotting, the data show freeze-mediated reductions (*p* < 0.05) in seven permissive histone modifications (H3K27me1, H4K20me1, H3K9ac, H3K14ac, H3K27ac, H4K8ac, H3R26me2a). Along with the maintenance of various repressive marks, the data are indicative of a suppressed transcriptional state at subzero temperatures. Elevated nuclear levels of histone H4, but not histone H3, were also observed in response to both cold and freeze acclimation. Together, the present study provides evidence for epigenetic-mediated transcriptional suppression in support of the winter diapause state and freeze tolerance of *E. solidaginis*.

## 1. Introduction

Many organisms are faced with seasonal environmental fluctuations that can jeopardize their survival. For numerous ectotherms (reptiles, amphibians, insects, etc.) that live in the Northern Hemisphere, cold hardiness and metabolic rate depression (MRD) are common necessities for enduring the winter months. Characteristics of these hypometabolic states include (1) the suppression of energetic demands, (2) reprioritized metabolic stores towards vital cellular processes and (3) the preservation of essential macromolecules through various protective mechanisms [1,2,3]. During bouts of hypometabolism, energetic resources are limited toward normothermic biological functions such as cell division, ion pumping, digestion, and macromolecule turnover. Costly subcellular processes such as transcription and translation are also commonly suppressed, with good reason. Gene transcription alone can account for upwards of 10% of the cellular energy budget, while similar studies have found protein synthesis to be even more demanding (~20–40% of ATP turnover depending on cell type) [4,5]. Indeed, a wide body of empirical evidence has implicated gene regulatory controls, notably epigenetic regulation, in the adaptation to stress of a diverse array of organisms [6,7,8,9,10]. Research supporting roles for epigenetic mechanisms in vertebrate stress responses is growing rapidly, whereas studies of most invertebrates are lagging behind, warranting a need in the current literature.

**Figure 1:**
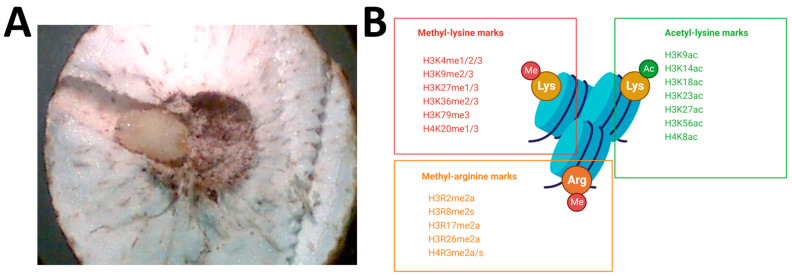
(**A**) Cross-sectioned goldenrod gall containing a third instar *E. solidaginis* larva sampled in early fall. Photo by Jan Storey. (**B**) Overview of studied histone H3 and H4 modifications. Created with BioRender.com.

The present study focuses on the phenomenon of freeze tolerance, a crucial winter survival strategy for many terrestrial ectotherms, especially among insects [11]. Many insects can reduce their metabolic output to <10% of normal during the winter months [12,13], often as a part of controlled metabolic rate depression in a phenomenon known as diapause. Diapause incorporates halted development and a physiological state of dormancy, typically occurring at a species-specific stage (e.g., egg, larva, pupa or adult), in response to adverse environmental conditions [14]. Insect species that overwinter in cold climates employ different cryoprotection strategies. Some use freeze avoidance, characterized by extremely high levels of cryoprotectants (mainly sugar alcohols) and the synthesis of antifreeze proteins [15]. Other species have evolved freeze tolerance, characterized by high levels of sugar alcohols to protect intracellular fluids from freezing, while using ice nucleating proteins to regulate controlled freezing in extracellular fluids, and other small molecules (e.g., trehalose, proline) to stabilize lipid bilayers to maintain the integrity of cell membranes [11,16]. The goldenrod gall fly, *Eurosta solidaginis* (Diptera: Tephritidae), is a well-researched model of freeze tolerance. Flies emerge from their galls in the spring, mate, and then females lay eggs in the newly growing stems of goldenrod (*Solidago* sp.). After hatching, each larva triggers the growth of a ball gall on the stem, within which it lives and eats. Third instar larvae overwinter (Figure 1A), pupate in the spring, and then the nonfeeding adults emerge to repeat the cycle. During the autumn, larvae use their large glycogen stores to synthesize cryoprotectants (mainly glycerol and sorbitol) that protect intracellular macromolecules and provide resistance to the loss of too much water into extracellular ice crystals [17]. Due to the stiff stems of the goldenrod plant, many galls project above the snow pack all winter and larvae must endure temperatures as low as −30 °C or more. Many of the underlying biochemical mechanisms driving gall fly freeze tolerance have been uncovered, although most of these studies revolved around metabolism, oxidative stress and protein stability [18,19,20,21].

Epigenetic mechanisms describe dynamic, heritable changes to functional chromatin that control the expression of nearby genes. Epigenetic mechanisms can be triggered by a plethora of stimuli that include but are not limited to cellular responses to stress, disease states, diet, exercise and development [22,23,24]. DNA methylation and histone modifications are two well-characterized classes of epigenetic regulation that have strong links to transcriptional gene regulation. The prevalence and function of DNA methylation in insect genomes is highly variable, with some studies suggesting that flies (order Diptera) lack widespread genomic methylation due to DNMT1/3 deletions [25,26,27]. Histone post-translational modifications (PTMs), and their conservation in flies, are much less controversial. Research on histone PTMs and their modifying enzymes in *Drosophila melanogaster* is extensive (reviewed in [28]). The present study was designed to analyze the role of histone PTMs in supporting the long-term freezing survival of goldenrod gall flies over the winter months.

Histones are responsible for the organized compaction of large amounts of genetic material as chromatin into eukaryotic nuclei. Histones are small, highly conserved, basic proteins that form the fundamental unit of chromatin, the nucleosome. Nucleosomes are roughly 150 base pairs of genomic DNA wrapped twice around an octamer of pairs of core histone proteins (H2A, H2B, H3 and H4), linked together by histone H1. Interestingly, N-terminal tails of histones protrude outward from nucleosomes and are subject to various PTMs that can have distinct regulatory effects on nearby gene transcription through chromatin remodeling. The best characterized reversible histone modifications to date are (1) arginine methylation, (2) lysine methylation and (3) lysine acetylation [29], although others such as phosphorylation, SUMOylation, ubiquitination and citrullination have been identified [30].

Methyl-arginine exists in three forms based on the position of the methyl groups: monomethylarginine (Rme), symmetric dimethylarginine (Rme2s) or asymmetric dimethylarginine (Rme2a). These are catalyzed by the nine members of the protein arginine methyltransferase (PRMT) family. The methylation of several N-terminal tail histone arginine residues by PRMTs has been associated with transcriptional regulation through chromatin immunoprecipitation (ChIP-Seq) experiments [31]. Permissive epigenetic marks include PRMT5-mediated symmetric dimethylation of H3R2, asymmetric dimethylation of H3R17/R26 by PRMT4 as well as PRMT1-mediated H4R3me2a. On the other hand, PRMT5-mediated H3R8me2s and H4R3me2s, as well as asymmetric dimethylation of H3R2 by PRMT6, are strongly associated with transcriptional repression [32]. Similar to arginine, lysine residues on histones tails can be either mono-, di- or trimethylated by “writer” lysine methyltransferases (KMTs) and demethylated by “eraser” lysine demethylases (KDMs). Comprehensive ChIP-Seq experiments have linked particular methyl-lysine modifications to transcriptional control [33,34]. There are six well-characterized histone lysine residues that undergo reversible methylation in chromatin—five on H3 (H3K4, H3K9, H3K27, H3K36, H3K79) and one on H4 (H4K20). Generally, methylated H3K4 (at promoters/enhancers) and H3K36 (at gene bodies) are enriched in active genes whereas methylated H3K9 and H3K27 colocalize with silenced genes through the action of heterochromatin protein 1 (HP1) isotypes and Polycomb repressive complexes (PRC), respectively [35]. In some cases, the extent of methylation (me1 vs. me3) tends to decide the functional significance of these modifications. For example, H4K20me1 is enriched within euchromatin to facilitate the transcription of housekeeping genes, whereas H4K20me3 plays a role in heterochromatin formation [36]. Finally, lysine-rich histone proteins are commonly acetylated by lysine acetyltransferase (KAT) enzymes from acetyl-CoA substrates, creating a strong link between metabolic and epigenetic transcriptional programming [37]. Histone acetylation is associated with transcriptionally permissive euchromatin formation via (1) locally relaxing the tight histone–DNA electrostatic interactions and (2) providing recognition sites for acetyl-binding proteins, mainly bromodomain (BRDs) proteins. These BRDs recruit chromatin remodeling complexes and/or transcriptional machinery [38]. Notable acetylated histone lysine residues include H3K9, H3K14, H3K18, H3K23, H3K27, H3K56 and H4K8. A summary of investigated histone modifications is found in Figure 1B.

The present study screened the global levels of prominent histone PTMs in goldenrod gall fly larvae with respect to cold (5 °C **→** −5 °C) and freezing (−5 °C **→** −15 °C) temperatures, in an attempt to link specific modifications to the hypometabolic freeze tolerance response of the insect.

## 2. Results

**Figure 2:**
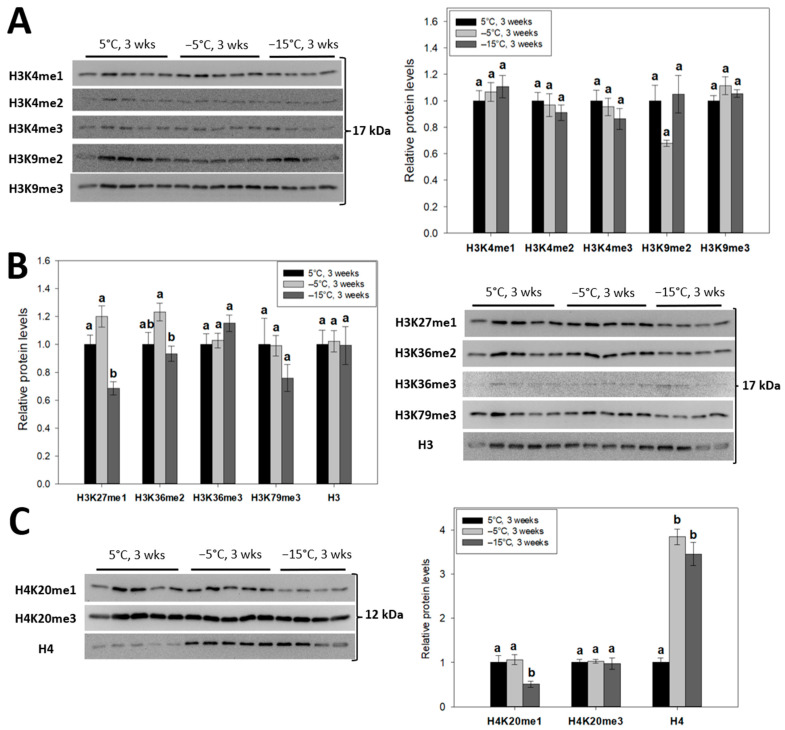
Relative levels of notable (**A**) H3K4/K9, (**B**) H3K27/K36/K79 and (**C**) H4K20 methyl-lysine modifications in *E. solidaginis* nuclear extracts from control, cold- and freezing-exposed larvae as determined by Western immunoblotting. Total histone H3 and H4 are also shown. Histograms display mean values ± SEM for *n* = 4–5 biological replicates from control (5 °C for 3 weeks), cold (−5 °C for 3 weeks) and frozen (−15 °C for 3 weeks) gall fly larvae. Data were analyzed using an analysis of variance (ANOVA) with a post-hoc Tukey’s test (*p* < 0.05). Values represented by the same letter are not significantly different from each other. H3K27me3 antibodies did not cross-react with nuclear extracts.

**Figure 3:**
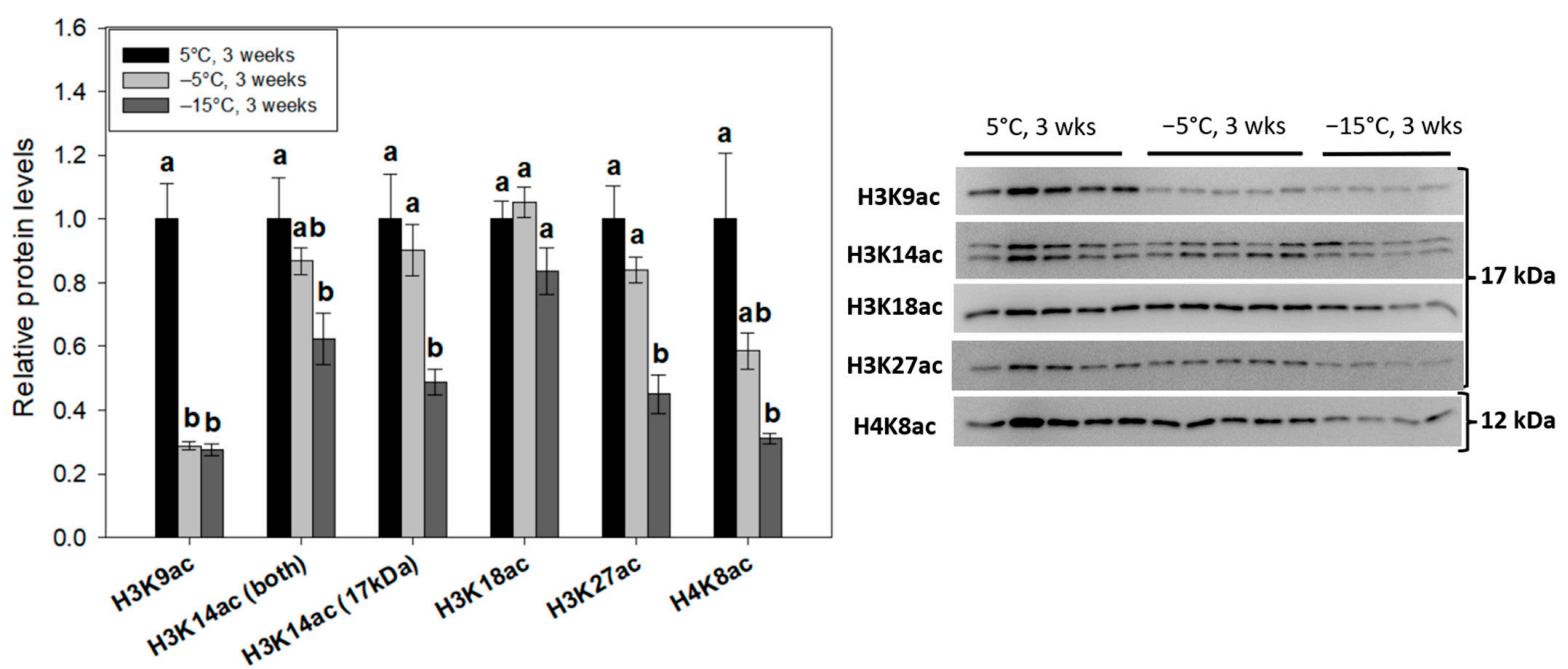
Relative levels of notable histone acetyl-lysine modifications in nuclear extracts of control, cold- and freezing-exposed *E. solidaginis* larvae as determined by Western immunoblotting. Histograms display mean values ± SEM for *n* = 4–5 biological replicates from control (5 °C for 3 weeks), cold (−5 °C for 3 weeks) and frozen (−15 °C for 3 weeks) gall fly larvae. Data were analyzed using an analysis of variance (ANOVA) with a post-hoc Tukey’s test (*p* < 0.05). Values represented by the same letter are not significantly different from each other. H3K23ac antibodies did not cross-react with nuclear extracts.

**Figure 4:**
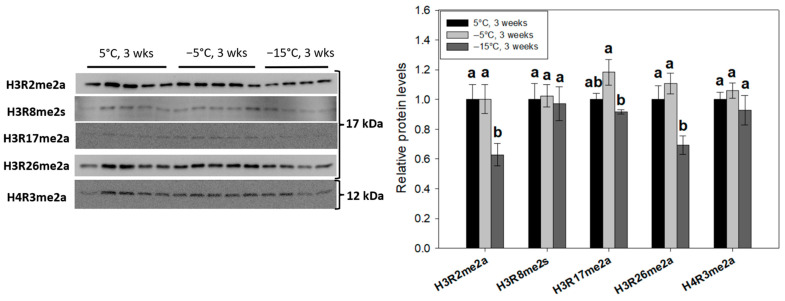
Relative levels of notable histone methyl-arginine modifications in *E. solidaginis* nuclear extracts from control, cold- and freezing-exposed larvae as determined by Western immunoblotting. Histograms display mean values ± SEM for *n* = 4–5 biological replicates from control (5 °C for 3 weeks), cold (−5 °C for 3 weeks) and frozen (−15 °C for 3 weeks) gall fly larvae. Data were analyzed using an analysis of variance (ANOVA) with a post-hoc Tukey’s test (*p* < 0.05). Values represented by the same letter are not significantly different from each other. H4R3me2s antibodies did not cross-react with nuclear extracts.

To profile changes in global histone H3/H4 modifications with respect to *E. solidaginis* freeze tolerance, immunoblotting was performed on nuclear extracts from larvae (*n* = 4–5 biological replicates) following prolonged (3 week) exposures to progressively lower temperatures (5 °C, −5 °C and −15 °C). Notable histone PTMs within three highly characterized classes—methyl-lysine (Figure 2A–C), acetyl-lysine (Figure 3) and methyl-arginine (Figure 4)—were tracked across the three treatment groups. Nuclear histone H3 and histone H4 protein levels were also assessed.

### 2.1. Permissive Monomethylations of H3K27 and H4K20 Are Significantly Reduced in Frozen Goldenrod Gall Fly Larvae

The relative abundance of 12 transcriptionally relevant histone methyl-lysine modifications were tracked in 3-week acclimated control (5 °C) larvae, cold-acclimated (−5 °C) larvae and frozen (−15 °C) larvae. One-way ANOVA with post-hoc Tukey’s tests were used to identify significant (*p* < 0.05) reductions in global levels of H3K27me1 (to 68.5 ± 5% of controls; Figure 2B) and H4K20me1 (to 50.5 ± 6% of controls; Figure 2C) in frozen larvae relative to the other treatment groups. A significant (*p* < 0.05) decrease in the relative abundance of H3K36me2 was also found during the transition from −5 °C to −15 °C. However, there were no significant (*p* < 0.05) changes in the levels of any of the H3K4 or H3K9 modifications investigated (Figure 2A) under cold or freezing stress (H3K9me2; *p* = 0.093 from controls).

### 2.2. Cold and Freezing Stresses Upregulate the Nuclear Levels of Histone H4, but Not H3

Along with their modifications, we followed the relative nuclear protein levels of total histone H3 and H4 with decreasing temperature exposures of the larvae. Using immunoblotting, levels of histone H3 were found to be consistent across treatment groups (Figure 2B). By contrast, strong cold- and freeze-mediated enrichment of nuclear histone H4 levels were found, rising by 3.8 ± 0.2-fold and 3.5 ± 0.3-fold, respectively (Figure 2C).

### 2.3. Decreased Acetylation of Prominent Histone Lysine Residues Occurs during Freezing

Histone acetylation levels changed significantly in response to larval freezing. Of the six acetyl-lysine modifications tested, four of them (H3K9ac, H3K14ac, H3K27ac, H4K8ac) were strongly reduced in response to larval freezing (*p* < 0.05; Figure 3). Global H3K9ac levels dropped to ~28% of control values following prolonged −5 °C exposure and remained low under freezing conditions at −15 °C. The relative abundance of H3K27ac and H4K8ac was also strongly reduced at −15 °C (0.45 ± 0.06 and 0.31 ± 0.01 of controls, respectively). Oddly, H3K14ac immunoblots produced a double band (slightly above and below 17 kDa in size), but regardless, analysis of either both bands or the lower band (which aligns with other H3 modifications) yielded a significant (*p* < 0.05) reduction at −15 °C relative to 5 °C controls. The upper band may be due to H3 ubiquitination. H3K18ac was the only histone lysine acetyl mark that was unresponsive to cold temperatures in gall fly larvae (*p* = 0.061; Figure 3).

### 2.4. H3R2me2a, H3R17me2a and H3R26me2a Are Cold-Responsive Methyl-Arginine Histone Modifications

Our initial screening of histone methyl-arginine marks tracked the levels of six notable modifications. We observed similar reductions (*p* < 0.05) in two asymmetrically methylated marks, H3R2me2a and H3R26me2a, with levels falling to 62.7 ± 7% and 69.3 ± 6% of controls, respectively (Figure 4). A significant decrease (*p* < 0.05) in nuclear H3R17me2a levels was also observed between −5 °C and −15 °C. However, the prevalence of H3R8me2s and H4R3me2a was unchanged across the three treatments (Figure 4).

## 3. Discussion

Freeze tolerance is a hypometabolic adaptive strategy employed by numerous ectothermic animals in response to freezing environmental temperatures. Underlying subcellular mechanisms of freeze tolerance continue to be discovered, which include controlled extracellular ice nucleation, intracellular accumulations of sugar cryoprotectants and MRD [17,39]. Protein synthesis is a major ATP-utilizing process suppressed in support of MRD through transcriptional and translational controls [4,5,40]. Recently, epigenetic regulation has been implicated in vertebrate freeze tolerance responses, particularly via histone PTMs [6,41,42]. Due to the high conservation of histone modification and their functions across eukaryotic life [28], and the need for epigenetic research of invertebrate hypometabolic responses, we investigated the prevalence of transcriptionally relevant histone PTMs during cold and freezing responses of *E. solidaginis* larvae.

### 3.1. Freeze Exposure Triggers Reductions in Many Transcriptionally Permissive Histone Modifications

The present study used immunoblotting of nuclear extracts from *E. solidaginis* larvae to profile the global levels of various histone PTMs and elucidate the transcriptional state of larval cells under decreasing environmental temperatures (5 °C, −5 °C and −15 °C) that mimic their seasonal exposure to falling temperatures and implementation of freeze tolerance. Our initial screen of 24 methyl-lysine, acetyl-lysine or methyl-arginine modifications found 10 of these to be significantly cold-responsive (*p* < 0.05) (Figure 2, Figure 3 and Figure 4). Notably, we observed eight modifications (H3K27me1, H4K20me1, H3K9ac, H3K14ac, H3K27ac, H4K8ac, H3R2me2a and H3R26me2a) whose prevalence on chromatin was significantly reduced under frozen (−15 °C, 3 weeks) conditions relative to controls (5 °C, 3 weeks). All of these modifications, except for one (H3R2me2a), are strongly associated with sites of active gene transcription, providing evidence in favor of transcriptional suppression in gall fly larvae during freezing.

Frozen gall fly larvae displayed reductions in permissive methyl-lysine H3K27me1 (Figure 2B) and H4K20me1 (Figure 2C) chromatin levels (68.5 ± 5% and 50.5 ± 6% of controls, respectively). In mammalian and *Drosophila* genomes alike, H3K27me1 localizes to transcriptionally active gene bodies [33,43] and has been shown to promote gene transcription [44]. Interestingly, H3K27me1 depletion has been similarly observed as part of the hypometabolic responses of freeze-tolerant wood frogs and anoxia-tolerant red-eared slider turtles [41,45]. In insects, H3K27me1 is generated by the opposing activities of PRC2 methyltransferase complexes and Jumonji-C domain UTX demethylases [35]. Hence, future studies into the roles of PRC2 and UTX during *E. solidaginis* freeze tolerance is warranted. H4K20me1, like H3K27me1, is distributed at actively transcribed intragenic regions [36], but unlike H3K27me1, it is heavily regulated by the cell cycle through an evolutionarily conserved mechanism. Chromatin H4K20me1 levels accumulate during late S phase due to stabilization of its sole monomethyltransferase, SETD8 [46]. Interestingly, knockdown studies of SETD8 and H4K20me1 trigger cell cycle arrest of *Drosophila* cells at late S phase [47]. Evidence for the arrest of cell proliferation has been suggested previously as a supportive hypometabolic mechanism [48,49] that may involve decreasing H4K20me1 in *E. solidaginis* during the quiescent state imposed by both diapause and freezing at low temperatures.

The acetylation of histone lysine residues stimulates local gene transcription through relaxing ionic histone–DNA interactions and recruiting bromodomain “readers” that associate with transcriptional machinery and/or permissive chromatin remodelers [38]. Not surprisingly, acetyl-histone levels were depressed for most investigated marks (H3K9ac, H3K14ac, H3K27ac, H4K8ac) following prolonged larval freezing (Figure 3). This is strongly indicative of heterochromatic structures and a suppressed transcriptional state, and aligns with *Eurosta* epigenetic research during shorter freezing timepoints [50]. MRD-mediated reductions in histone acetyl marks (particularly H3K9ac and H3K23ac) have been observed recently, primarily in animal responses to anoxia [7,51] and during ground squirrel hibernation, both being hypometabolic states [8,52]. These studies suggest the changes are due to heightened deacetylase action [7] and lowered nuclear acetyltransferase activity [53]. In mammalian cells, histone acetylation is directly linked to the acetyl-CoA synthetase, ATP-citrate lyase (ACL) [54], that breaks down carbohydrate-derived citrate to produce cytoplasmic/nuclear pools of acetyl-CoA that can then be used for fatty acid biosynthesis [55]. ACL activity is greatly reduced in *Eurosta* larva during overwintering [56], and this makes sense since sugar reserves need to be maintained as cryoprotectants and are largely unmetabolized during the freeze. A link between ACL activity, nuclear acetyl-CoA availability and histone acetylation levels in *E. solidaginis* freeze tolerance is underexplored.

ChIP-seq experiments with *Drosophila melanogaster* have highlighted active gene promoters that are characterized by increased levels of H3K9ac, H3K27ac and H3K4me3 [57]. Hence, the depletion of H3K9ac and H3K27ac (to ~28% and ~45% of control values; Figure 3) along with the maintenance of repressive modifications (H3K9me3, H4K20me3) during freezing are indicative of repressed global transcription. H3K9ac tends to co-occur with H3K14ac at both active euchromatic and heterochromatic regions [58,59], whereas H3K27ac antagonizes PRC2 gene silencing activities in *Drosophila* [60]. H4K8ac may play a key in role in transcriptional elongation rather than initiation, as it recruits the SWI/SNF complex, but not RNA Pol II machinery [61]. In frozen *E. solidaginis,* the reduction in H4K8ac (to ~31% of controls) and modest reduction in H3K14ac levels (62.3 ± 8%), combined with depleted H3K9ac/H3K27ac, likely work in conjunction to repress both transcriptional initiation and elongation processes.

Our profile of methyl-arginine modifications highlighted two marks of varied function that were significantly reduced in response to prolonged freezing (Figure 4): H3R2me2a (62.7 ± 7%) and H3R26me2a (69.3 ± 6%). H3R2me2a is typically associated with gene silencing via preventing the deposition of a permissive H3K4me3 mark on the adjacent residue in eukaryotic genomes [62]. Interestingly, decreasing H3R2me2a prevalence did not correlate with elevated global H3K4me3 levels (Figure 2A) in the frozen state. Separate ChIP-seq experiments show that H3R2me2a is enriched at pericentromeric regions of human chromosomes, and oddly is most abundant within highly expressed genes [63]. Another study found repressive transcriptional effects of H3R2me2a at gene promoters, but permissive effects at enhancers [64]. These authors concluded that the presence of H3R2me2a can severely disrupt nearby histone PTMs. Here, it is likely that decreased H3R2me2a plays a previously undiscovered role in the overwintering state of Eurosta third instar larvae, where it may exhibit either repressive and/or permissive transcriptional functions. It is also possible that its depletion in frozen *E. solidaginis* may “prime” larval genomes for permissive modifications (such as H3K4me3) to induce transcription upon thawing. In mammalian systems, H3R26me2a and H3R17me2a are common substrates of PRMT4 (CARM1), a major transcriptional coactivator of gene promoters [31]. Agreeably, the levels of H3R26me2a and H3R17me2a under decreasing larval temperatures correlate well, with both marks displaying significant (*p* < 0.05) reductions from −5 °C to −15 °C (Figure 4). The *Drosophila* PRMT1 and PRMT4 homologues (DART1/4) have the highest sequence similarities with their mammalian counterparts (84% and 72%) of all arginine methyltransferases [65]. Furthermore, the substrate specificities of DART1/4 are highly similar to PRMT1/4, suggesting conserved mechanisms in insects. Recent research on another freeze-tolerant animal, the wood frog *Rana sylvatica*, demonstrated ~5-fold reductions in both PRMT4 expression and Type I (asymmetric dimethylation) PRMT activity in the liver of frozen frogs, an effect that was reversed upon thawing [42]. All in all, our data suggest that decreases in chromatin H3R26me2a and H3R17me2a may play a role in suppressing global gene transcription in support of hypometabolism in frozen *Eurosta* larvae, and this may be due to repressed DART4 (PRMT4).

### 3.2. Elevated Levels of Histone H4 May Play a Role in Transcriptional Regulation and Cell Protection

An unexpected finding of this study was the cold-responsive nature of histone H4, but not histone H3. Histone H4 levels rose sharply by 3.8 ± 0.2-fold in nuclear samples from −5 °C acclimated larvae, as compared with controls, and were maintained in larvae frozen at −15 °C (3.5 ± 0.3-fold; Figure 2C). This effect was not matched by histone H3, whose expression was unaffected across the tested treatment groups (Figure 2B). Coincidently, similar trends were observed in studies of the brains of frozen wood frogs [6]. How elevated expression of histone H4 contributes to metabolic rate depression and freeze tolerance of *E. solidaginis* is unknown, but interesting nonetheless. Some evidence has highlighted roles for changes in histone availability as a gene regulatory control [66], whereas other studies have described antioxidant and antiapoptotic functions of H4 in cancer cells [67], although implicating these mechanisms is highly speculative at this point.

### 3.3. Conclusions

We tracked the relative abundance of 24 histone H3/H4 PTMs in goldenrod gall fly third instar larvae following prolonged (3 week) exposures to cold (5 °C **→** −5 °C) and freezing (−5 °C **→** −15 °C) temperatures. Ten modifications were significantly (*p* < 0.05) responsive to decreasing temperatures. Freeze exposure triggered reductions in seven permissive (H3K27me1, H4K20me1, H3K9ac, H3K14ac, H3K27ac, H4K8ac, H3R26me2a) and one repressive (H3R2me2a) histone PTM. Interestingly, histone H4 expression, but not H3, was induced under cold and freezing conditions. The present findings support the growing evidence for epigenetic-mediated transcriptional suppression in support of animal freeze tolerance and suggest that there might be undiscovered similarities in the mechanisms of invertebrate and vertebrate systems alike.

## 4. Materials and Methods

### 4.1. Animal Experiments

Galls containing third instar larvae were collected from fields in the Ottawa (Ontario, Canada) area during early October, then stored in cardboard trays and acclimated at 5 °C in incubators for 3 weeks. A subset of sampled control *E. solidaginis* larvae (5 °C, 3 weeks) were then cut out of their galls and frozen immediately in liquid nitrogen. Incubator temperature was then dropped to −5 °C and remaining galls were held for a further 3 weeks (due to high cryoprotectant concentration, larvae do not freeze at this temperature). Cold-exposed (−5 °C, 3 weeks) animals were then sampled as above. Finally, temperature was further reduced to −15 °C (a temperature that freezes the larvae but sustains viability) and a third group of insects were frozen (−15 °C, 3 weeks) inside their galls followed by sampling after 3 weeks. All sampled larvae were immediately killed by immersion in liquid nitrogen and stored at −80 °C until use.

### 4.2. Nuclear Protein Isolation

Each biological replicate was a pool of 4–5 frozen goldenrod gall fly larvae (~100 mg) with *n* = 5 replicates per experimental condition. Larvae were quickly homogenized using a glass–glass Dounce homogenizer in 1:4 *w*/*v* cold 1× cytoplasmic protein buffer (10 mM HEPES, 10 mM KCl, 10 mM EDTA, 20 mM β-glycerophosphate, 1 mM dithiothreitol and 10 µL/mL protease inhibitor cocktail (BioShop, Cat# PIC001.1, Burlington, ON, Canada), pH 7.9). Homogenates were then centrifuged at 12,000× *g*, 4 °C for 15 min, and supernatants containing cytosolic protein were collected. Nuclear pellets were washed with cytoplasmic buffer followed by the addition of 250 µL of 1× nuclear protein buffer (20 mM HEPES, 400 mM NaCl, 1 mM EDTA, 10% *v*/*v* glycerol, 20 mM β-glycerophosphate, pH 7.9) and then sonicated. Samples were then centrifuged (10,000× *g*, 4 °C) for 15 min to separate nuclear protein from debris, and supernatants were collected. Protein concentrations of soluble cytosolic and nuclear protein were quantified using the BioRad protein assay (BioRad Laboratories, Cat# 5000002, Hercules, CA, USA). Concentrations were standardized to 5 μg/μL with respective fraction buffers and then mixed 1:1 *v*/*v* with 2× SDS buffer (100 mM Tris-base, 4% *w*/*v* SDS, 20% *v*/*v* glycerol, 0.2% *w*/*v* bromophenol blue, 10% *v*/*v* 2-mercaptoethanol). Protein samples were denatured in a boiling water bath for 10 min and then immediately placed on ice. Effective fractionation was verified by immunoblotting to detect appropriate subcellular markers: cytoplasmic (α-tubulin) and nuclear (histone H3/H4). Samples were then stored at −40 °C until use.

### 4.3. Western Immunoblotting

Equal amounts of nuclear protein extract (5–15 μg) were loaded alongside BLUelf Prestained Protein Ladder (10–245 kDa; FroggaBio, Cat# PM008-0500, Concord, ON, Canada) in discontinuous SDS-polyacrylamide gels. Upper stacking gels were 5% acrylamide:bis-acrylaminde *v*/*v* in 1 M Tris buffer (pH 6.8), 0.1% SDS, 0.1% APS and 0.1% TEMED. Lower separating gels were 15% acrylamide:bis-acrylamide *v*/*v* in 1.5 M Tris buffer (pH 8.8), 0.1% SDS, 0.1% APS and 0.1% TEMED. Nuclear protein was separated by gel electrophoresis using the BioRad Mini Protean III system (BioRad Laboratories, Hercules, CA, USA) with 1× Tris-glycine running buffer (25 mM Tris-base, 190 mM glycine, 0.1% *w*/*v* SDS, pH 8.5) at 150 V for ~60 min. Separated protein samples were then electroblotted by wet transfer onto 0.45 micron polyvinylidene difluoride (PVDF) membranes for 45 min at 100 mA in 1× Tris-glycine transfer buffer (25 mM Tris-base, 190 mM glycine, 10% *v*/*v* methanol, pH 8.5).

Following transfer, PVDF membranes were blocked by rocking in 5% *w*/*v* skim milk in 1× TBST (20 mM Tris-base, 140 mM NaCl, 0.05% Tween-20) for 30 min at room temperature. Membranes were washed 3 × 5 min in 1× TBST, before rocking overnight (~18 h) at 4 °C in the presence of histone-modification-specific anti-rabbit primary antibodies (diluted 1:1000 *v*/*v* in 1× TBST, 0.02% *w*/*v* sodium azide). The next day, membranes were washed 3 × 5 min in 1× TBST, then rocked for 30 min at room temperature with 1:8000 *v*/*v* HRP-linked goat anti-rabbit secondary antibodies (BioShop, Cat# APA007P.2, Burlington, ON, Canada) in 1× TBST. Membranes were then washed for 3 × 5 min in 1× TBST before immunoblot bands were imaged by enhanced chemiluminescence (H_2_O_2_ and Luminol) using a ChemiGenius Bio Imaging System (Syngene, Frederick, MD, USA). Representative whole Western immunoblot images can be found in the Supplementary Materials. ECL band intensities were standardized against representative Coomassie-stained (0.25% *w*/*v* Coomassie brilliant blue, 7.5% *v*/*v* acetic acid, 50% methanol) total nuclear protein in the same lane for loading controls.

Antibodies used in this study were purchased from commercial companies and are as follows:

ABclonal Technology (Woburn, MA, USA): H3K4me2, A2356; H3K4me3, A2357; H3K9me2, A2359; H3K27me1, A2361; H3K27me3, A2363; H3K36me3, A2366; H3K79me3, A2369; H4K20me1, A2370; H4K20me3, A2372; H3K14ac, A7254; H3K18ac, A7257; H3K27ac, A7253; H3K56ac, A7256; H3R17me2a, A2421; H3R26me2a, A2375; H4R3me2a, A2376; Histone H3, A2348; and Histone H4, A17024.

Abcam (Cambridge, UK): H3K4me1, ab8895; and H3K9me3, ab8898.

Cell Signaling Technology (Danvers, MA, USA): H3K9ac, #9649P; H3K23ac, #9674S; and H4K8ac, #2594P.

Active Motif (Carlsbad, CA, USA): H3K36me2, #39256.

MyBioSource (San Diego, CA, USA): H3R2me2a, MBS9402172; H3R8me2s, MBS9607605; and H4R3me2s, MBS126222.

### 4.4. Quantification and Statistics

Enhanced chemiluminescent band intensities were quantified by densitometry using a ChemiGenius Bio Imaging System and GeneTools Software (version 4.3.8.0, Syngene, Frederick, MD, USA). To account for loading controls, bands were standardized against representative total nuclear protein of Coomassie-stained bands separated from the histone band range (10–20 kDa). Histogram data are mean ± SEM for *n* = 4–5 samples from different pools of *E. solidaginis* larvae. Statistical analyses were conducted by one-way ANOVA and post-hoc Tukey’s tests (*p* < 0.05) using SigmaPlot 12.0 statistical software (Systat Software Inc., San Jose, CA, USA).

## Data Availability

Representative full immunoblot images are attached in a supplemental file alongside this manuscript. The data that support the findings of this study are available from the corresponding author upon reasonable request.

## Supplementary Materials

The supporting information can be downloaded at https://www.mdpi.com/article/10.3390/ijms241210153/s1.
