# Supplementary material for: Histone H3 and H4 Modifications Point to Transcriptional Suppression as a Component of Winter Freeze Tolerance in the Gall Fly Eurosta solidaginis
Source: Int J Mol Sci. 2023 Jun 15;24(12):10153. doi: 10.3390/ijms241210153 (PMC10299125; doi:10.3390/ijms241210153)
Supplement: Supplementary file 1 [file ijms-24-10153-s001.zip › ijms-2430869-supplementary.pdf]

Representative whole Western immunoblot images

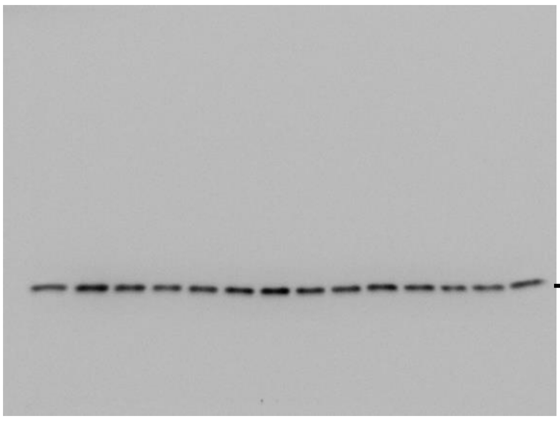

H3K4me1
17 kDa

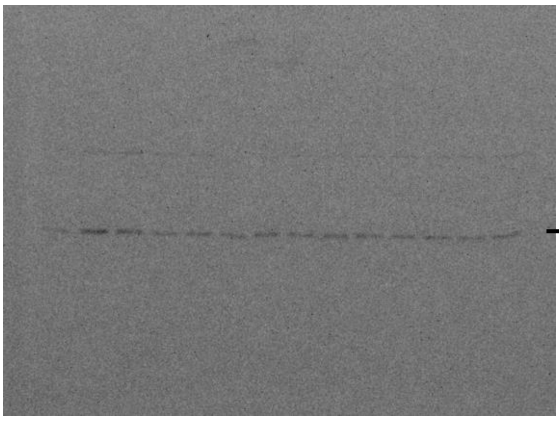

H3K4me2
17 kDa

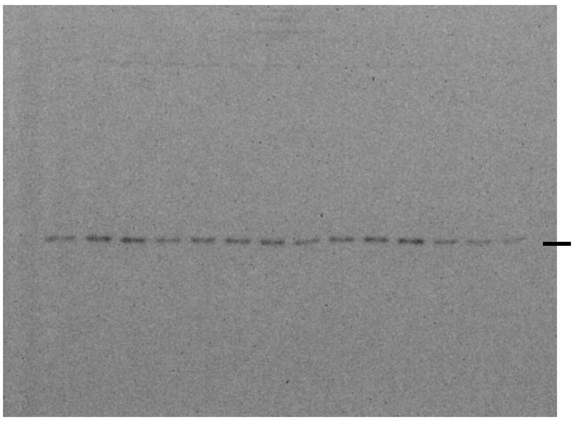

H3K4me3
17 kDa

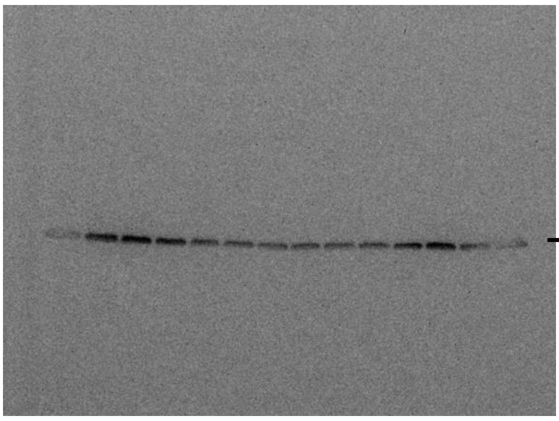

H3K9me2
17 kDa

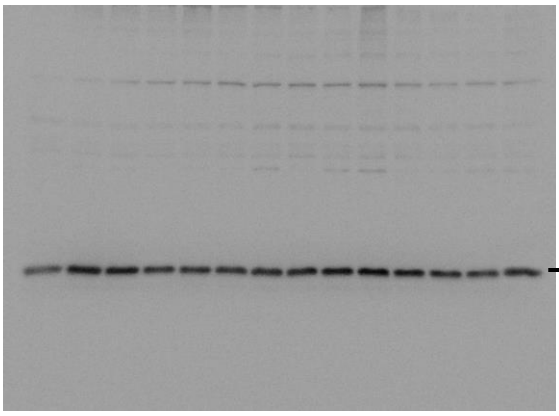

H3K9me3
17 kDa

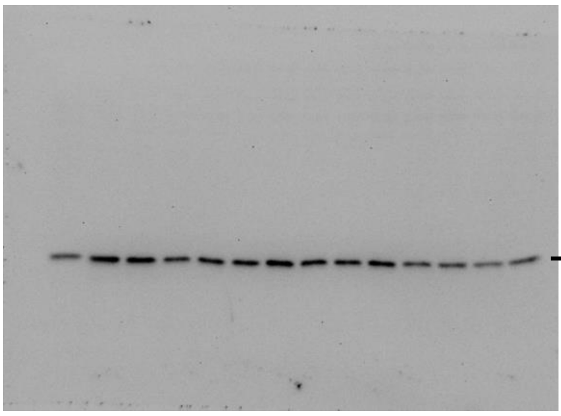

H3K27me1
17 kDa

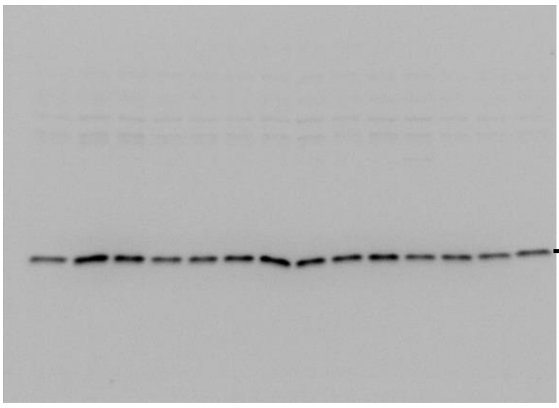

H3K36me2
17 kDa

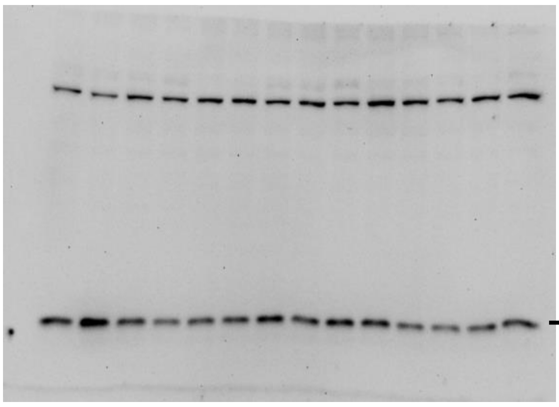

~50 kDa

H3K79me2
17 kDa

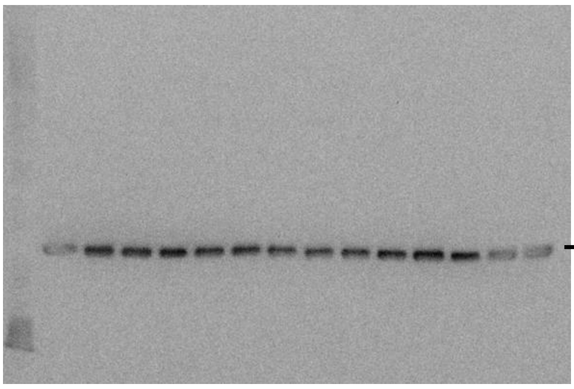

H3
17 kDa

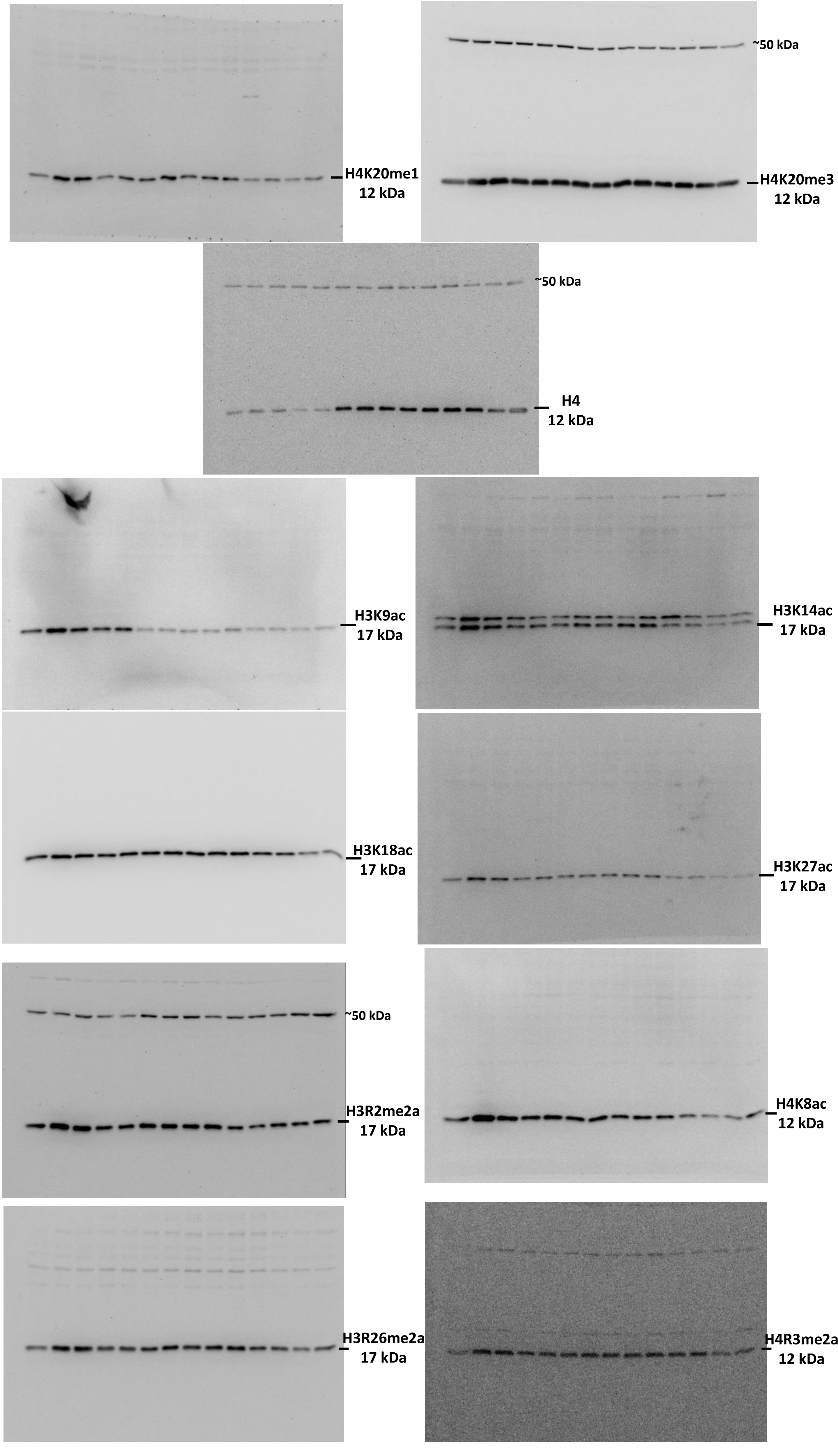
H4K20me1
12 kDa
~50 kDa
H4K20me3
12 kDa
~50 kDa
H4
12 kDa
H3K9ac
17 kDa
H3K14ac
17 kDa
H3K18ac
17 kDa
H3K27ac
17 kDa
~50 kDa
H3R2me2a
17 kDa
H4K8ac
12 kDa
H3R26me2a
17 kDa
H4R3me2a
12 kDa
